# A Data-Driven Adaptive Emotion Recognition Model for College Students Using an Improved Multifeature Deep Neural Network Technology

**DOI:** 10.1155/2022/1343358

**Published:** 2022-05-26

**Authors:** Li Liu, Yunfeng Ji, Yun Gao, Tao Li, Wei Xu

**Affiliations:** ^1^Jiangsu Vocational College of Information Technology, Wuxi, Jiangsu 214153, China; ^2^Jiangsu Key Laboratory of Media Design and Software Technology (Jiangnan University), Wuxi 214122, China

## Abstract

With the increasing pressure on college students in terms of study, work, emotion, and life, the emotional changes of college students are becoming more and more obvious. For college student management workers, if they can accurately grasp the emotional state of each college student in all aspects of the whole process, it will be of great help to student management work. The traditional way to understand students' emotions at a certain stage is mostly through chats, questionnaires, and other methods. However, data collection in this way is time-consuming and labor-intensive, and the authenticity of the collected data cannot be guaranteed because students will lie out of impatience or unwillingness to reveal their true emotions. In order to explore an accurate and efficient emotion recognition method for college students, more objective physiological data are used for emotion recognition research. Since emotion is generated by the central nervous system of the human brain, EEG signals directly reflect the electrophysiological activity of the brain. Therefore, in the field of emotion recognition based on physiological signals, EEG signals are favored due to their ability to intuitively respond to emotions. Therefore, a deep neural network (DNN) is used to classify the collected emotional EEG data and obtain the emotional state of college students according to the classification results. Considering that different features can represent different information of the original data, in order to express the original EEG data information as comprehensively as possible, various features of the EEG are first extracted. Second, feature fusion is performed on multiple features using the autosklearn model integration technique. Third, the fused features are input to the DNN, resulting in the final classification result. The experimental results show that the method has certain advantages in public datasets, and the accuracy of emotion recognition exceeds 88%. This proves the used emotion recognition is feasible to be applied in real life.

## 1. Introduction

With the development of the times, the environment in which college students live is also constantly changing. The transformation of the identity of college students is accelerating, and they are faced with problems in study, work, interpersonal communication, and life. Most of the current college students were born after 1995. They have a strong sense of independence, have their own ideas, and are emotionally sensitive. In addition, in the current summer of the epidemic, students' learning and employment have been greatly affected. The epidemic not only affects the physical health of college students but also affects their emotions. On the one hand, when college students surf the Internet, they will be confronted with a variety of epidemic-related information, which will have a psychological impact. On the other hand, college students need to adapt to the comprehensive online teaching state and complete various learning tasks arranged by the school. College students will experience stressful emotions for an extended period of time under these conditions, and negative emotional states such as loneliness, anxiety, depression, and fear will emerge. If you do not pay attention and adjust in a timely manner, it may result in more serious consequences. As a result, whether it is college students or colleges and universities, they should pay close attention to the emotional state and mental health of college students while focusing on the epidemic and their studies.

For college student management workers, the traditional methods to understand students' emotions mainly include interviews, questionnaires, and oral presentations by class informants. The main problems in these methods are as follows: first, some students are worried that telling the truth will cause adverse effects and other reasons to falsely report information, resulting in inaccurate information collected. Second, some students are disgusted or impatient to fill in the questionnaire, so they fill in the content at will, resulting in inaccurate information collected. Third, some students' emotions are not exposed, so it is difficult to obtain accurate information through the observation of classmates and teachers. Therefore, in order to obtain accurate and effective emotional data, it is best to obtain it based on objective physiological data. Since the emotion recognition results based on physiological signals are more objective, various emotion recognition methods based on physiological signals emerge as the times require. These physiological signals are noninvasive in nature, easily obtained from the individual, and largely reflect the influence of emotion on the automatic nervous system. Electrocardiogram (ECG) [1], electromyography (EMG) [2], ElectroSkin (GSR) [3], respiration rate (RR) [4], and EEG [5] are all commonly used for emotion recognition. Physiological signals: in particular, EEG signals are most often used for emotion recognition [6, 7]. EEG signals have been widely used to study swallowing, analyze mental states, and assist in diagnosing neuropsychiatric diseases [8–10].

EEG-based emotion recognition research is mainly carried out from the following aspects. one is the research based on the recognition model, mainly based on machine learning algorithms and based on deep learning algorithms. Reference [11] applies Support Vector Machine (SVM) to binary classification tasks or to few classifications and achieves good results. In SVM, by calculating the distance between each support vector, a maximum interval space is optimized to realize the distinction of different categories [12]. Reference [13] annotates the feature information of the ECG signal according to the local features and overall features of the ECG signal, and SVM is used to identify the ECG. However, when the data scale becomes large, using SVM for classification will consume a lot of machine memory and computing time. Reference [14] classified four kinds of ECG signals through a decision tree ensemble algorithm, and the classification Fl-score exceeded 0.81. Reference [15] uses a one-dimensional convolutional neural network to perform feature extraction on ECG signals, and the accuracy rate reaches 86% in the ECG four-classification task. Reference [16] proposes to combine a convolutional neural network (CNN) with a recurrent neural network (RNN) to achieve a multiscale representation of ECG signals. However, due to the limited depth, the ECG signal feature extraction is incomplete, and the improvement of the classification effect is not obvious. Reference [17] extracted a set of ECG signal features to describe the morphological features of the entire ECG signal and improved the classification accuracy based on XGBoost. Reference [18] used DNN to classify 12 kinds of signals in single-lead ECG signal, and its classification performance accuracy was as high as 83.7%. The second is research based on different emotion expression models. There are mainly continuous emotional expression models that represent emotions, mainly two-dimensional [19], three-dimensional [20], 4-dimensional [21], and discrete emotional expression models [22–24]. The third is based on different EEG feature extraction. Methods of research include the power spectrum feature [25] and wavelet energy entropy feature [26].

At present, EEG-based emotion recognition is mainly carried out from the above three aspects. Multifeature extraction strategies are mostly combined with machine learning algorithms for research. Most of the research on deep learning algorithms focuses on improving the algorithm itself. Inspired by this, this study combines a multifeature fusion strategy with a deep learning algorithm to further increase the emotional EEG′ recognition accuracy. Considering that different features can represent different information from the original data, in order to express the original EEG data information as comprehensively as possible, various features of the EEG are first extracted. Second, feature fusion is performed on multiple features using the autosklearn model integration technique. Third, the fused features are input to the DNN, resulting in the final classification result. The experimental results show that the method has certain advantages in public datasets, and the accuracy of emotion recognition exceeds 88%. This proves that the used emotion recognition method is feasible to be applied in real life.

## 2. Relevant Background Knowledge

### 2.1. Emotional Characteristics of College Students

1. Anxiety and loss due to employment and higher education pressure: in addition to academic pressure, some senior students feel anxious when faced with the pressure of employment and further education. In order to protect the health of college students, the government requires college students not to return to school and colleges to reopen until the epidemic is effectively controlled. This means that many recent university graduates cannot improve their employability through internships. Due to the severe epidemic situation, many companies have not resumed work in time or have not resumed normal production and operations after the resumption of work, and corporate recruitment will inevitably be affected. Affected by this, college students who are applying for jobs generally show negative emotions such as anxiety and loss.
2. Under the epidemic environment, online learning at home has led to burnout and anxiety. Affected by the epidemic, colleges and universities have delayed the start of school, and domestic colleges and universities have adopted online teaching methods to enable college students to study courses at home. The school arranges a week's class schedule according to the students' course selection, and the teacher selects the appropriate online platform for teaching and assignments according to the needs of the course. Many college students are taking online classes for the first time. Coupled with the influence of factors such as irregular work and rest during the holidays, they have a lot of discomfort with online teaching, which in turn has an impact on their emotional state. Some students expressed their rejection of online classes and even thought of dropping out. Each teacher has different requirements and needs to use many software programs such as QQ, WeChat, and Tencent Conference. Due to problems such as network or software capacity, there are often problems such as freezes and flashbacks during class, which affect the status of the class. Teachers are worried about the effect of students studying at home, and they will assign more homework. Students feel that they are either taking online classes or rushing to do homework every day and feel exhausted.
3. Sadness and anger caused by following all kinds of news: sudden emergencies will greatly stimulate the public's information exchange and dissemination behavior. With the help of Internet technology, the spread of epidemic-related information has become very fast. But for the recipient of the information, it can be difficult to discern which is credible and which is a rumour from the dizzying array of information. The prevention and control of the epidemic require the public to reduce going out. College students respond to the government's call to stay at home for a long time, and they can only obtain information through TV news, online media, and various social software. College students should be concerned about current affairs, but 24-hour reports on the epidemic, closed home isolation environment, etc. will make individuals pay too much attention to the epidemic. In addition, there are some negative news on the Internet that are exaggerated to attract attention. If you are too immersed in negative social information, you will have negative emotions that are not good for your physical and mental health.

### 2.2. Emotion Expression Model

The representation of discrete emotions is derived from basic emotion theory. The basic emotion theory divides emotion into basic emotion and secondary emotion. Basic emotion is determined by the human physiological autonomic nervous system. Assuming that there are various neural channels in the human body system, each basic emotion corresponds to its own functional channel and presents certain physiological feedback to the surrounding environment and situation. Such feedback may be physiological electrical signals, body movements, voice intonation, natural language expressions, and facial expressions. Secondary emotion is no longer restricted by basic emotion; it can reflect individualized emotion through the combination of different basic emotions or the fusion of cognition and inner needs. There are three typical evaluation criteria for discrete emotions, namely: Scherer affective state model [27], OCC affective model [28], and Roseman affective model [29].

Some scholars believe that emotional states do not exist in isolation but are distributed in multiple dimensional spaces. Therefore, any two affective states in a continuous affective space can be brought together by the continuity of dimensions. Emotions that are closer in space have a greater similarity. Conversely, the farther the distance in the space is, the smaller the similarity is, and the easier it is to distinguish. In addition, the continuous emotional space can also be measured by Euclidean distance, and the similarity of emotions can be quantified according to the distance, which makes it easier to analyze the emotional state. Typical continuous emotional expression models include Wundt emotional space [30], Schlosberg 3D cone emotional space [31], 3D emotional space for PAD [32], and Plutchik emotional wheel continuous space [33]. The relationship between the above two types of emotion expression models is not completely independent but an inclusive relationship. Continuous types of emotion models include discrete types of emotion expression models. Moreover, even within a certain type of emotion expression model, there is an inclusive relationship between several typical emotion expression models.

### 2.3. EEG Features

Before the EEG raw data are input to the classifier, it needs to perform preprocessing, feature extraction, and dimensionality reduction. Based on preprocessed EEG data such as denoising, multidomain feature extraction is required. Generally, effective features are extracted from the time domain, frequency domain, and spatial domain, respectively. A brief introduction to the features of each domain is shown in Table 1.

## 3. Emotion Recognition Models

### 3.1. Emotion Recognition Architecture

**Figure 1:**
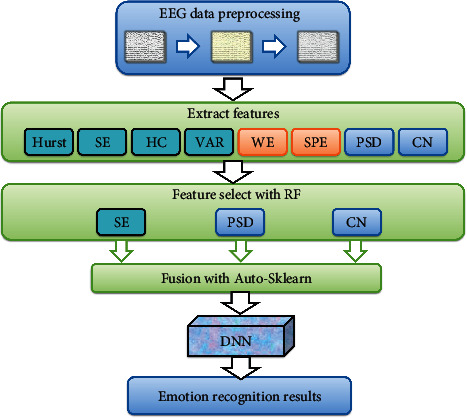
Emotion recognition architecture diagram.

In order to maximize the use of feature data in various fields of EEG, the classification performance of the model can be improved as much as possible. This article extracts 8 features in the three domains of time domain, frequency domain, and spatial domain given in Section 2.3. The eight features are Hurst, sample entropy (SE), Hjorth complexity (HC), vector autoregression (VAR), wavelet entropy (WE), spectral entropy (SE), power spectral density (PSD), and complex network (CN). Although the increase of feature dimension can convey the features of EEG more comprehensively and objectively, the number of redundant and invalid features it contains also increases. While increasing the computational complexity, it also reduces the classification accuracy. Therefore, feature selection is very necessary. Feature selection is to filter out the most effective feature subsets from the original features. Model fusion aggregates the classification results of many models to improve single-model performance. Under the assumption that a single model produces uncorrelated errors, deep learning models based on multidomain features may allow incorrect models to be rejected in decision-making. Based on DNN, this article proposes a DNN model that combines multifeature input and applies it to the recognition of emotion EEG.  Figure 1 illustrates the architecture of the used recognition method.

As shown in Figure 1, firstly, eight features are extracted from the input raw EEG data. Second, the importance of the features is sorted using a random forest (RF) algorithm, and the top 3 most important features are selected. Third, use the autosklearn model integration technology to perform feature fusion on the three features; fourth, input the fused features into the DNN to obtain the final classification result.

### 3.2. Feature Selection Strategy

When filtering feature information based on RF, the importance value (IV) of all feature information can be calculated to sort the features according to their importance. Taking a decision tree *i* as the initial point, based on equation (1), the out-of-bag data error (*OD*_*error*_) is obtained. After that, the value of the feature information *H*^*j*^ in the out-of-bag data is rearranged, keeping other eigenvalues unchanged. Then a new out-of-bag data set can be obtained, denoted as *OD*_*error'*_. Similarly, use equation (1) to obtain a new out-of-bag data error (*OD*_*error*_). The IV of the feature information *F*^*j*^ in the ith decision tree can be derived by subtracting the results of the two calculations.

(1) $$\begin{array}{c} {\text{OD}}_{\text{error}}=\frac{1}{n}\sum_{i=1}^{n}{\left(x_{f}-x_{c}\right)}^{2}, \end{array}$$

where *n* represents the total amount of data, *x*_*f*_ represents the actual value of data, and *x*_*c*_ represents the classification value of data.

(2) $$\begin{array}{c} \text{IV}\left(H^{j}\right)={\text{OD}}_{{\text{error}}_{i}^{\prime }}-{\text{OD}}_{{\text{error}}_{i}}. \end{array}$$

According to the same principle, the corresponding IV is obtained for each decision tree in the random forest, and finally all the obtained IV are averaged to obtain the IV of the feature information *H*^*j*^:

(3) $$\begin{array}{c} \text{IV}\left(H^{j}\right)=\frac{1}{m}\sum_{\text{i}=1}^{m}{\text{IV}}_{i}\left(H^{j}\right), \end{array}$$

where *m* is the number of decision trees in random forests. It can be seen that if feature information is very important, its numerical value varies greatly among different samples. When the feature values in the out-of-bag dataset are reordered, the difference between different samples will be reduced so that *OD*_*error*_ will increase. Then according to (2) and (3), it can be concluded that the larger the IV of the feature information, the stronger its importance.

By calculating the IV of all feature information, combined with the reverse search method of the sequence, a set of optimal prediction feature information subsets can be obtained. Considering the high dimension of the track feature information, if the reverse search method of the sequence is used directly, the operation time will be too long, thus reducing the operability of the algorithm. Therefore, this article uses an improved sequence reverse search algorithm. Specifically, a rough selection stage is added before the reverse search method is applied to the feature information set. Specific steps are as follows:

**Step 1 .**

. Train the random forest using the extracted eight kinds of feature data. After the training is completed, *IV* of all feature information can be obtained. At this time, the test set is used to evaluate the classification accuracy *e*_*all*_ of the random forest algorithm, and this value is set as the classification threshold.

**Step 2 .**

. Sort the *IV* of all feature information in descending order. After the sorting is completed, the feature information corresponding to the first 15 *IV* is imported into the rough selection feature information set *S*_pre_, which is initially an empty set, and this feature information is deleted from the initial feature set *K* at the same time.

**Step 3 .**

. The RF is retrained based on *S*_pre_^*i*^, and the classification error e_pre_^*i*^ of the test is obtained. The upper right corner mark *i* in the formula represents the number of feature information of the information set *S*_pre_.

**Step 4 .**

. Determine the size relationship of *e*_pre_^*i*^. If *e*_pre_^*i*^ ≥ *e*_all_^*i*^, then add 15 more feature information to the feature information set *S*_pre_^*i*^ to form a new set *S*_pre_^*i*+15^. If *e*_pre_^*i*+15^ ≤ *e*_pre_^*i*^, do not add feature information to *S*_pre_; otherwise, continue to select the top 15 feature information in *K* to add.

After the rough selection information set *S*_pre_ is determined, the reverse sequence search method is used for this set. The IV corresponding to all feature information in set *S*_*pre*_ are sorted from small to large, and then a feature information variable corresponding to the smallest IV is eliminated. The RF is trained again based on this set, and the classification results in the test set are saved.

After the above operations are completed, the feature information variable corresponding to the next IV is eliminated, and training, testing, and recording are repeated until the initial set *S*_pre_ becomes an empty set. Finally, considering the classification accuracy of each scheme comprehensively, determine the appropriate feature information set *S*_best_.

## 4. Experimental Simulation and Discussion

### 4.1. Feature Selection Experiment

**Figure 2:**
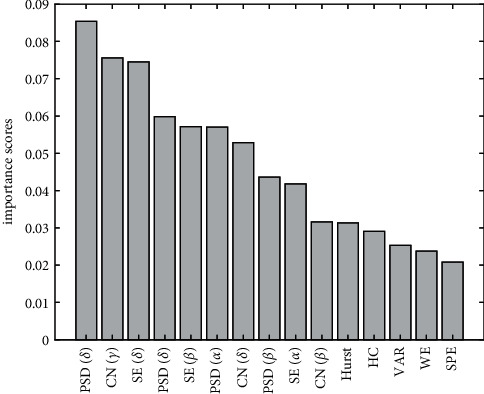
Feature sorting.

The PI value corresponding to each feature information is calculated based on RF and arranged in descending order. This selects features that play an important role in the classification decision-making process. The number of populations in RF is 500 by default. In order to better compare the importance of each feature, the calculated PI value is normalized to represent the importance of each feature.  Figure 2 gives the top 15 features based on RF.

It can be seen from Figure 2 that the feature importance of each frequency band of the PSD ranks first, indicating that its importance is the greatest. It is speculated that the classification results of PSD in CNN may be good, so PSD is selected as one of the optimal feature subsets. After the PSD is the CN, and after the CN is the SE. Behind SE is Hurst. Considering that the more the features are, the longer the model training takes, and the classification results are not necessarily good. Therefore, this article finally selects the top 3 features, namely PSD, CN, and SE.

### 4.2. Model Evaluation Experiment

The dataset used in this article is the public DEAP dataset. The tenfold cross-validation strategy was adopted, and 70% of the EEG sample set was used as the training set to build the emotion recognition model. Use the established emotion recognition model to test the remaining 30% of the test samples to perform emotion recognition. The comparison models include SVM [34], CNN [35], DNN [36], RNN [37], LSTM [38], [39], and [40], and the parameter settings of each comparison model are consistent with those in the article. To make the model converge quickly, the Adam optimizer employs the adaptive learning rate method so this model employs the Adam optimizer, the initial learning rate is set to 0.001, the Dropout layer ratio is set to 0.2, and the batch size is set to 32. The epoch is 80, and for each epoch, a full training process is performed on all training sets.  Table 2 depicts the experimental environment for this article. The accuracy rate, precision rate, and F1 score are the quantitative indicators used to assess the model's effectiveness. The three indicators' calculation formulas are as follows:

(4) $$\begin{array}{c} \text{Accuracy}=\frac{\text{TP}+\text{TN}}{\text{TP}+\text{TN}+\text{FP}+\text{FN}},\\ \text{Precision}=\frac{\text{TP}}{\text{TP}+\text{FP}},\\ \text{Recall}=\frac{\text{TP}}{\text{TP}+\text{FN}},\\ F1=\frac{2*\text{Precision}*\text{Recall}}{\left(\text{Precision}+\text{Recall}\right)}. \end{array}$$

**Figure 3:**
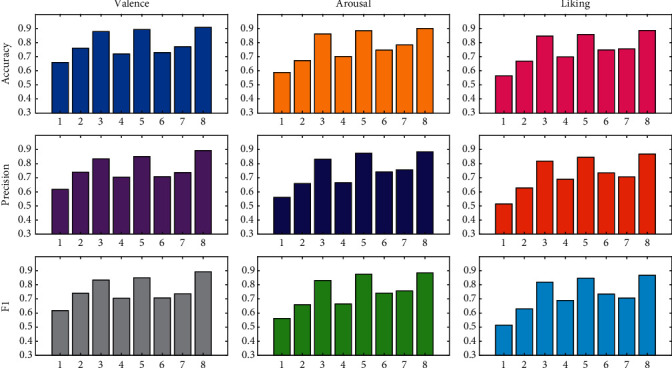
Comparison of experimental results.

The experimental results of each algorithm on the DEAP dataset are shown in Table 3. Since the presentation method in the form of a table cannot visually show the small gap between the numbers, this article compares and displays the experimental data of each method on different indicators and different emotional dimensions in the form of legends, as shown in Figure 3.

The above simulation data illustrate that the overall recognition effect on the Valence dimension is the best, followed by Arousal, and the worst is Liking. Comparing the recognition results of each model, first of all, the machine learning representative algorithm SVM performs the worst, which shows that for EEG-based classification tasks, the deep learning algorithm has more advantages in terms of recognition accuracy. Second, comparing each deep learning model, the classification accuracy of each model is between 0.7 and 0.9. CNN, RNN, [39], and [40] are all below 0.8. The classification performance of LSTM is good. This is due to the fact that, despite being an RNN variant model that inherits most of the RNN model's characteristics, LSTM solves the vanishing gradient problem caused by the gradual reduction of the gradient backpropagation process, thereby improving model classification performance. The recognition accuracy of DNN has risen to more than 0.8. This demonstrates that increasing the number of network layers can improve the model's classification accuracy. Based on DNN, the model in this article has increased Accuracy, Precision, and *F*1 by 3.48%, 7.05%, and 9.07%, respectively, in the Valence dimension. In the Arousal dimension, Accuracy, Precision, and *F*1 increased by 4.6%, 6.31%, and 2.44%, respectively. In the Liking dimension, Accuracy, Precision, and *F*1 increased by 4.82%, 6.05%, and 2.1%, respectively. It can be seen that no matter which dimension it is, the recognition effect of the model in this paper is improved by more than 4 points compared with DNN. This fully demonstrates the effectiveness of this method in processing the input feature data.

**Figure 4:**
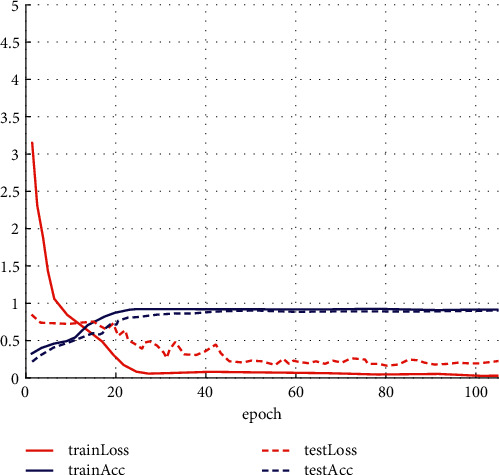
Loss and accuracy of training and test sets in Valence dimension.

This experiment records the classification accuracy and loss of the proposed method in the Valence dimension of the training set and the test set at different epochs.  Figure 4 illustrates that when the training round reaches about 25 in the training set, the model gradually tends to be stable. At this time, the loss value is reduced to 0.082, and the accuracy rate reaches 0.9124. In the test set, when the number of training rounds reaches about 40, the model gradually stabilizes. At this time, the loss value is 0.2633, and the accuracy reaches 0.9078.

**Figure 5:**
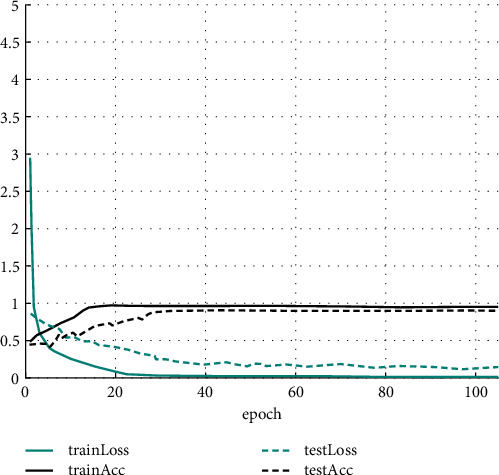
Loss and accuracy of training and test sets in the Arousal dimension.

The experiment also records the classification accuracy and loss of the model in the training set and the test set in the Arousal dimension under different epochs.  Figure 5 shows when the training round reaches about 20 in the training set, the model gradually tends to be stable. At this point, the loss value is 0.086, and the accuracy is 94.79%. In the test set, when the number of training rounds reaches about 30, the model gradually stabilizes. At this time, the loss value is 0.1703, and the accuracy reaches 90.21%.

Figures 4 and 5 show that the training set's classification accuracy and model convergence speed are greater than those of the test set. However, there is still overfitting, which has an impact on the final classification effect. Therefore, the improvement of the overfitting phenomenon will also be the content that needs further research in this article in the future. It can be expanded by increasing the amount of data, regular terms, or reducing the complexity of the model.

## 5. Conclusion

The identity and characteristics of college students are not the same as other students. They are relatively independent and have a certain rudimentary understanding of society and their own responsibilities. They are under increasing pressure in their studies, life, work, and other aspects as society and technology develop. College students' mental health is receiving increasing attention. The emergence of the epidemic in recent years has caused the global economy to suffer. Employment has dropped sharply, and college students' job-hunting and living pressures have intensified. Multiple internal and external pressures make the number of students with mental health problems increase year by year. If students with psychological problems can be identified as soon as possible and given timely and appropriate psychological counseling and help, many tragedies can be reduced. In order to detect students with mental health problems as early as possible, it is necessary to grasp the psychological state of each classmate as accurately as possible and be very clear about the emotional changes of students. Therefore, this article proposes an emotion recognition method based on EEG data. Considering that different features can represent different information from the original data, in order to express the original EEG data information as comprehensively as possible, various features of the EEG are first extracted. Second, use the autosklearn model integration technology to perform feature fusion on the screened features. Third, input the fused features into the DNN to get the final classification result. The experimental results demonstrate that the method in this article has a good effect on the emotion recognition of college students. The method shows certain advantages in public datasets, and the accuracy of emotion recognition exceeds 88%. The advantage of this method is that the recognition results based on physiological data are more accurate, and the recognition results are close to 90%, which can be applied in real life. However, there is still room for further improvement in this study, such as continuing to improve the recognition accuracy and how to easily collect EEG data in real life. The emotion recognition results of college students should not be limited to the application of mental health but can also be extended to classroom teaching. In the process of classroom teaching, if teachers can obtain real-time emotional changes in students, they can better promote the improvement of teaching methods. For example, when the teacher explains a certain knowledge point, some students are obviously anxious, indicating that these students do not understand the content of the teaching, then the teacher can change the teaching method at this time. When the teacher explained a certain knowledge point, the students' emotion recognition results were happy and positive, indicating that the students were very interested in this part of the content. Through the emotion recognition results, the students' learning situation can be sensed in real time in the classroom, and teachers can dynamically adjust their teaching methods and teaching progress according to the obtained results. On the other hand, although the EEG-based emotion recognition system is accurate, the data collection is inconvenient. Therefore, it is also important to develop a collection device that is convenient, fast, and low-cost and does not affect students' classroom learning.

## Figures and Tables

**Table 1:** EEG features.

Domain	Feature
Time domain	Skewness, kurtosis, zero-crossing rate, instability index, Hurst's index
	Detrended fluctuation analysis, Pearson's fractal dimension, sample entropy,
	HFD, Hjorth activity, hjorth mobility, hjorth complexity
	Energy, RMS, vector autoregression (VAR)

Frequency domain	Power, wavelet entropy, spectral entropy, PSD, partial directed coherence (PDC)
	Band power (BP)

Spatial domain	Index of asymmetry, complex network (CN)

**Table 2:** Description of experimental software and hardware environment.

Name	Details	Name	Details
CPU	I9 9900 K	Editor	PyCharm COMMUNITY 2018.3
RAM	32G DDR4 3200 MHz	Locales	Python 3.6
GPU	GTX 1070	Deep learning framework	Tensorflow 1.12
Graphics card	NVIDIA GeForce RTX2080	Operating system	Windows10

**Table 3:** Experimental results of each model on different emotional dimensions.

Model	Index	Valence	Arousal	Liking
SVM	Accuracy	0.6587	0.5882	0.5643
	Precision	0.6174	0.5612	0.5144
	*F*1	0.5693	0.5610	0.4757

CNN	Accuracy	0.7596	0.6716	0.6685
	Precision	0.7396	0.6586	0.6285
	*F*1	0.7264	0.6659	0.6217

DNN	Accuracy	0.8773	0.8606	0.8457
	Precision	0.8338	0.8316	0.8182
	*F*1	0.8163	0.8573	0.8383

RNN	Accuracy	0.7196	0.7016	0.6985
	Precision	0.7047	0.6644	0.6891
	*F*1	0.7160	0.6254	0.6850

LSTM	Accuracy	0.8924	0.8841	0.8572
	Precision	0.8501	0.8744	0.8459
	*F*1	0.8346	0.8282	0.8244

Reference [39]	Accuracy	0.7288	0.7469	0.7475
	Precision	0.7069	0.7413	0.7346
	*F*1	0.6767	0.7769	0.7235

Reference [40]	Accuracy	0.7697	0.7830	0.7550
	Precision	0.7367	0.7571	0.7064
	*F*1	0.7583	0.7983	0.7022

Proposed	Accuracy	0.9078	0.9002	0.8865
	Precision	0.8926	0.8841	0.8677
	*F*1	0.8903	0.8782	0.8559

## Data Availability

The labeled dataset used to support the findings of this study is available from the corresponding author upon request.
